# Data sharing in clinical trials: An experience with two large cancer screening trials

**DOI:** 10.1371/journal.pmed.1002304

**Published:** 2017-05-23

**Authors:** Claire S. Zhu, Paul F. Pinsky, James E. Moler, Andrew Kukwa, Jerome Mabie, Joshua M. Rathmell, Tom Riley, Philip C. Prorok, Christine D. Berg

**Affiliations:** 1 Division of Cancer Prevention, National Cancer Institute, Bethesda, Maryland, United States of America; 2 Information Management Services, Inc., Rockville, Maryland, United States of America; 3 Division of Cancer Epidemiology and Genetics, National Cancer Institute, Bethesda, Maryland, United States of America

## Abstract

Paul Pinsky of the US National Cancer Institute and colleagues describe the implementation and outcomes of web-based data sharing from the PLCO and NLST cancer screening trials.

**Summary points**

- Broad sharing of clinical trial data is important for ensuring reproducibility, transparency, and maximal use of the data by the research community. However, in practice, such data sharing typically requires planning, effort, and resources.
- Here, we describe a web-based data sharing system, the Cancer Data Access System (CDAS), developed for two large cancer screening trials: the Prostate, Lung, Colorectal and Ovarian (PLCO) cancer screening trial and the National Lung Screening Trial (NLST).
- Deidentified individual participant data were organized into standard datasets readily downloadable from CDAS via a simple web-based application process that involves minimal scientific review. CDAS provides a “one-stop shop” for access requests, review, and data downloads.
- Since the launch of CDAS in November 2012 and through October 2016, 215 requests were received for PLCO data, of which 199 (93%) were approved, and 240 requests were received for NLST, of which 216 (90%) were approved.
- The estimated cost of CDAS was around US$300,000 for the initial development, plus additional maintenance and user-support costs of about US$26,000 per month. Because of its modular nature, additional studies can be added to CDAS with relatively little additional cost.

## Introduction

Over the last several years, the idea of sharing data from clinical trials has been much discussed, especially for government-funded research. In 2015, the Institute of Medicine (now the National Academy of Medicine) published guiding principles and a framework for the responsible sharing of clinical trial data [1]. While journals have long been requesting submitters to deposit high-throughput molecular data in public databases, such requirements have not been systematically applied to clinical trial data. Recently, however, the International Committee of Medical Journal Editors (ICMJE) issued proposed requirements for the sharing of data generated from interventional clinical trials as a condition for publication in member journals [2]. Specifically, the proposed requirement stated that “the authors will be required to share the de-identified individual-patient data (IPD) underlying the results presented in the article no later than 6 months after publication”.

Currently, a number of platforms and initiatives exist for the broad sharing of clinical trial data. The National Heart, Lung, and Blood Institute (NHLBI) of the National Institutes of Health (NIH) established a data-sharing platform, the Biologic Specimen and Data Repository Information Coordinating Center (BioLINCC), in 2000 for access to data and biospecimens from NHLBI-funded studies [3]. In recent years, major pharmaceutical companies have begun sharing their clinical trial data with the broad scientific community through web-based systems. Examples are the Yale Open Data Access (YODA) project, ClinicalStudyDataRequest.com, and Supporting Open Access to Researchers (SOAR) [4–6]. The Project Data Sphere initiative is another new data-sharing platform focused on data from Phase III cancer clinical trials [7].

However, to date there has been relatively little in the literature describing in detail the experience with all facets of a specific clinical trial data-sharing initiative. A description of the costs, utilization, and logistics of such initiatives is useful in helping institutions determine the best options for their data-sharing needs and for funding entities, regulatory agencies, and other interested parties to assess the implications of proposed policies regarding data sharing. Here, we discuss our experience in data sharing for two large-scale screening trials conducted by the National Cancer Institute (NCI): The Prostate, Lung, Colorectal and Ovarian (PLCO) Cancer Screening Trial and the National Lung Screening Trial (NLST). To our knowledge, this is one of the earliest attempts at broad data sharing for any major cancer-related clinical trial.

## The PLCO and NLST trials

PLCO was a randomized trial testing the effectiveness of screening for prostate, lung, colorectal, and ovarian cancers. It enrolled almost 155,000 men and women aged 55–74 at ten screening centers from 1993 to 2001 and randomized them to a screening or usual care arm. Data were obtained on screening tests, diagnostic follow-up procedures, all-cancer incidence and characteristics, and mortality. PLCO also collected self-reported demographic, dietary, and medical history data. Follow-up continued for at least 13 years from randomization.

NLST was a randomized trial comparing low-dose computed tomography (LDCT) and chest radiographs for lung cancer screening in high-risk ever smokers. Almost 55,000 men and women aged 55–74 were randomized at 33 centers between 2002 and 2004. NLST collected data similar to PLCO’s data. Follow-up continued for up to 7 years from randomization.

The primary outcome for each PLCO component (prostate, lung, colorectal, and ovarian) was cancer-specific mortality. The main PLCO trial results were published from 2009 to 2012 [8–11]. The primary outcome for NLST was lung-cancer specific mortality, which was published in 2011 [12].

## The Cancer Data Access System

In 2011, after the primary outcome results of NLST and PLCO were reported or were soon to be reported, NCI decided to make the data from both trials publicly accessible, with minimal administrative burden and without any requirement for collaboration with a trial investigator. The decision was driven by the high public interest in the trials’ results and a desire for maximizing transparency about the trials’ findings.

To ensure broad access and efficient data transfer, it was decided that a web-based system would best serve this purpose. The NHLBI had already established such a system, BioLINCC, for sharing data and biospecimens collected from various NHLBI-supported clinical and epidemiological studies [3]. The goal was to develop a central hub for researchers to access deidentified individual participant data from PLCO and NLST and for managing and administering the application process. As the trials were initiated decades ago, the original consent forms did not include specific language for broad data sharing; however, the consent language does not specifically prohibit such sharing. An NCI institutional review board (IRB) reviewed the consent forms and approved the Cancer Data Access System (CDAS) project, under the stipulation that the available data would be compliant with the Health Insurance Portability and Accountability Act (HIPAA) Privacy Rule. Trial participants were not notified of the data-sharing initiative. Development of the system, denoted CDAS, was funded by an NCI contract and took approximately one year.

During the initial development phase, the following specific requirements were identified: (1) ensuring participant confidentiality; (2) creating documented and downloadable standardized datasets; (3) developing an online submission, review, approval, and data delivery system; and (4) developing a tracking and archiving system of approved requests and resulting publications. Below are details of the development processes for each of these requirements. Details on CDAS system requirements are given in S1 Text. The URL for CDAS is https://biometry.nci.nih.gov/cdas.

### Ensuring participant confidentiality

The “Safe Harbor” method was used to achieve deidentification in accordance with the HIPAA Privacy Rule (https://www.hhs.gov/hipaa/for-professionals/privacy/special-topics/de-identification/index.html#protected). Personally identifiable information (PII) and protected health information (PHI) were deleted. Self-reports of sexually transmitted diseases were excluded. Event dates were replaced with days since randomization. Various small cells (e.g., age at first pregnancy above 40) were collapsed into broader categories to prevent identification, as were some conditions that occurred relatively infrequently in the study population (e.g., cirrhosis and hepatitis into one liver comorbidity variable).

### Standardized datasets and data documentation

For PLCO, for each trial cancer, a main dataset and 5–7 auxiliary datasets were created (Table 1). Main datasets included most of the variables needed for standard analyses (e.g., demographics, screening and diagnostic follow-up results, cancer incidence and characteristics, and mortality). Auxiliary datasets provided additional details about screening results, diagnostic procedures, and cancer treatment.

**Table 1:** Data dictionary characteristics.

	PLCO: Prostate	PLCO: Lung	PLCO: Colorectal	PLCO: Ovarian	NLST
Customized datasets (main and auxiliary)	6	6	8	6	16
Variables in main dataset	221	211	454	218	260
Total unique variables in main and auxiliary datasets	324	373	711	383	410
Total data dictionary pages (main and auxiliary)	~70	~70	~100	~70	~75
Total file records	408,000	599,400	447,100	269,600	567,000

For each standardized dataset (main or auxiliary), a data dictionary was created containing the variable name, label, description and a code or format. Most of the variables needed to reproduce the findings from the major PLCO papers (covering the primary outcome and results of baseline and subsequent screening rounds) are in the main dataset, but some specific analyses may require auxiliary dataset variables.

Other standardized datasets were also created, including for nontrial cancers and ancillary studies. For genomic studies using PLCO biospecimens (e.g., genome-wide association studies, whole exome sequencing), investigators are required to deposit the data in the NIH database of Genotypes and Phenotypes (dbGaP) in accordance with the NIH Genomic Data Sharing Policy; these genomic data are widely available for secondary uses through dbGaP. Genomic data downloaded from dbGaP can then be merged with PLCO demographic and clinical data available through the CDAS. With respect to imaging data, digitized chest radiographs from the lung screening exams are available upon request.

For NLST, a main dataset and 15 auxiliary standardized datasets were created, with dataset construction similar to PLCO (Table 1). In addition to data, users can also request the low-dose CT screening exam images. These images (about 72,000) are stored in a separate database, The Cancer Imaging Archive (TCIA), which hosts a large archive of medical images for public download.

### Online request submission, review, approval, and dataset delivery

For each trial, the CDAS website includes a section providing background information (trial design, available data, questionnaires and study forms, past and ongoing research projects, and publications) and an online application submission module for requesting data. Requestors use the online submission module to submit their proposal, supplying project details including investigator identifying information, a short project summary with specific aims, and desired standardized datasets. Requestors can also communicate with CDAS staff, monitor application status, and modify existing requests.

The review and approval process is managed in CDAS, which includes functionalities for viewing and approving requests and monitoring approved projects. The entire request lifecycle is managed through CDAS. The NCI, with contract support, reviews requests for feasibility (i.e., whether the research questions can be addressed with available data) and clarity and interacts with requesters as needed. NCI may request revisions of the project summary or specific aims as appropriate (for example, if it is too vague). The review does not assess scientific merit or methodology, nor does it check for duplicate research projects.

After approval, a Data Transfer Agreement (DTA) is generated for each non-NIH institution included among approved users (S2 Text). Additional approved users, beyond the requestor, may gain permission for data access after project approval. Approved users and an authorized institutional signatory must sign and return the DTA, and the requestor must agree to have the project description publicly listed on CDAS before trial data can be accessed. The DTA specifies, in part, that the requestor will not attempt to identify trial participants, that the data will only be used for the proposed project, and that the requestor is required to notify CDAS of any resultant publications.

CDAS generates a compressed delivery package for each approved project that includes datasets, data dictionaries, and a user guide explaining the data and trial; the package is accessed through a web portal. On a case-by-case basis, custom datasets can be generated to include additional data, merge files, and define populations of interest. For NLST LDCT image requests, approved users can access the images and download them via TCIA or request a hard drive containing the images to be delivered via mail.

### Tracking and archiving of approved requests and resulting publications

Approved projects and publications are archived and linked in a searchable CDAS database. Search results can be filtered by keywords and publication year and exported to a spreadsheet. Search results for projects show a ranking of relevance for the search, project title and ID; investigator name and institution; and project request date. Publications are entered into the database after they are identified by investigators or found on PubMed; search results show the title, authors, journal, year, PubMed ID, and abstract.

## CDAS utilization

The NCI made various efforts to advertise the CDAS website and the availability of trial data; these efforts included announcements sent out to listservs of a number of relevant scientific organizations, talks at large cancer-related research conferences, and a scientific paper in a major cancer journal describing the overall PLCO research resource, which mentioned CDAS [13].

The CDAS website went live for applications for both NLST and PLCO in November 2012. From then through October 31, 2016 (48 months), 215 requests were submitted for PLCO data, of which 199 (93%) were approved. The main reason for nonapproval was because proposal aims were judged not feasible with available data. The average number of approved projects per month was 4.1. For NLST, of 240 requests submitted during the same period, 214 (89%) were approved; reasons for nonapproval were similar to those for PLCO. The average number approved per month was 4.5.

Table 2 lists investigator type for approved projects. In PLCO, approximately one-third were NCI researchers or associated with PLCO screening centers. In NLST, this fraction was around one-fifth. For NLST, a substantial fraction, 22%, were from the private sector, with the majority of these private sector investigators focused on computer-aided detection or diagnosis (CAD/CAD-X).

**Table 2:** Approved proposals by principal investigator affiliations.

	PLCO	NLST
	*n* (%)	*n* (%)
NCI (intramural and extramural)	59 (29.6)	25 (11.7)
Trial screening center	7 (3.5)	13 (6.1)
Other United States	113 (66.8)	120 (56.1)
Academic, government, or research Institute	106 (53.2)	90 (42.1)
Private sector	7 (3.5)	30 (14.1)
International	20 (10.1)	56 (26.2)
Academic, government, or research institute	19 (9.5)	38 (17.8)
Private sector	1 (0.5)	18 (8.4)
Total	199	214

Table 3 lists the research categories of approved projects. For PLCO, 33% were screening trial–related and 42% focused on cancer etiology. For NLST, 42% were trial-related and an additional 44% were focused on image analysis, primarily CAD/CAD-X of lung cancer or lung nodules using LDCT images.

**Table 3:** Approved proposals by subject area.

	PLCO	NLST
	*n* (%)	*n* (%)
Screening trial–related	66 (33.2)	90 (42.1)
Image analysis (CAD/CAD-X)	6 (3.0)	95 (44.4)
Cancer etiology	84 (42.2)	10 (4.7)
Risk prediction	14 (7.0)	5 (2.3)
Other	29 (14.6)	14 (6.5)
Total	199	214

We also analyzed publications resulting from approved CDAS projects. For this analysis, approved projects were matched with known trial publications based on author names, publication, proposal abstracts, and publication and CDAS request dates. A Kaplan–Meier analysis was performed for time until first publication of an article derived from the research proposal, with censoring at the end of follow-up (October 31, 2016).

For PLCO, through 3 years of follow-up from project initiation, 25% of projects resulted in publications as estimated by the Kaplan–Meier analysis (S1 Data). By principal investigator (PI) affiliation (NCI or trial center versus others), the proportions were 33% and 20%, respectively (*p* = 0.46, log-rank test). For NLST, the estimated proportion publishing within 3 years was 19% (S2 Data). Excluding image analysis projects, for which the goal was generally not publication (especially for those from the private sector), the 3-year proportion publishing was 27%. Overall, 31 PLCO and 21 NLST projects resulted in at least one publication to date.

## CDAS development and operating cost

The initial development cost of CDAS is estimated at around US$290,000, including web and database development, data preparation, and making the webpages Section 508 compliant. Ongoing operation and maintenance involves three primary components: (1) general website maintenance, (2) basic processing of CDAS requests, and (3) programming support for CDAS requests. Item 2 involves handling DTAs and routine communications with the applicant and NCI; item 3 involves working with applicants to identify data needs and creating customized datasets. Estimated monthly costs for these three items are US$1,000, US$5,000, and US$20,000, respectively.

The above costs do not include the development of data dictionaries and other trial documentation. Note that since most documentation was created and revised during the trial for the purposes of Data and Safety Monitoring Board reports and major trial publications, it is difficult to separate out these costs from those for CDAS per se.

Additionally, while not technically part of CDAS, there were costs associated with the storage and maintenance of the NLST LDCT images at TCIA, images that were generally accessed through CDAS. Annual costs for this were about US$215,000.

It should be noted that the above figures represent our best estimates of associated costs. With CDAS, and likely with other data sharing initiatives, true costs are difficult to assess. With CDAS, the same contractor had already developed the aforementioned and similar BioLINCC website, which CDAS built upon; therefore, the cost of developing CDAS would have been greater if not for this earlier effort. Secondly, it is difficult to separate costs for developing the data and image database as a resource for the trial investigators—which would have been done anyway, regardless of whether there was any broad data-sharing effort—from costs specific to broad data sharing. This is especially true with CDAS since the trial data are not static but are periodically being updated because of data cleanup, the receipt of new event data from ongoing follow-up of trial participants, and the addition of new ancillary data (e.g., assay results from biospecimen studies). As noted, about one-third (for PLCO) of projects were initiated by trial or NCI researchers. Although these projects did not require a broad data-sharing model, some infrastructure would still have been needed to prepare, document, and deliver the appropriate datasets to these researchers.

## CDAS expansion

The original purpose of CDAS was for sharing trial data from PLCO and NLST; however, from the outset, NCI recognized that the system should be designed such that other studies could be added with relatively little development effort. Such a design not only reduces costs associated with developing a database system from scratch but also provides a “one-stop-shop” for accessing multiple trials and/or studies at once. As such, CDAS was later made more extensible so that data and associated documentation for any new study could easily be added. This modification cost approximately US$130,000 and was implemented in one year. Following this modification, the Interactive Diet and Activity Tracking in AARP (IDATA) study was added to CDAS. This addition cost approximately US$17,000 and was implemented in 3 months.

## Comparisons with other data-sharing platforms

In contrast to CDAS, which includes data from two large clinical trials, most other data-sharing platforms contain data from many, generally smaller, trials. The NHLBI-supported BioLINCC currently contains data from 100 clinical trials, with a cumulative total of almost 350,000 participants [14]. BioLINCC began in 2000, with the number of available trials increasing steadily over time. A total of 370 requests were received from 2000–2016; 30% of requests were associated with at least one publication involving trial data. A recent analysis examined requests to three other open-access platforms for clinical trials: YODA, SOAR, and ClinicalStudyDataRequest.com [5]. Together, these platforms contain data on 3,255 clinical trials. From 2013 to 2015, 234 proposals were submitted, of which 154 were approved. Of all included trials, 15.5% were requested at least once. Project Data Sphere (PDS), a free digital library and data laboratory launched in 2014, currently contains data from 72 oncology trials representing over 41,000 study participants [15]. PDS has been used for crowdsourcing challenges, including one for predicting survival of men with advanced prostate cancer.

## Challenges and lessons learned

An important challenge in data sharing is data format and quality and clarity of data descriptions. PLCO and NLST data were collected in a standardized format and subjected to rigorous cleanup procedures; additionally, well-curated data dictionaries are available on CDAS as well as original trial questionnaires and data-collection forms. Further, the CDAS team included personnel familiar with trial data. CDAS personnel frequently interacted with requestors prior to project approval to advise on interpretation of trial variables and on which standard files were needed for their research question. Misinterpretation of trial data by secondary users has been noted as a concern in clinical trial data sharing [16]. The above-cited features of CDAS help to mitigate against this occurrence but do not entirely eliminate the risk.

Another potential limitation of data-sharing platforms is the seemingly low publication rate, which in CDAS is about 20%–25%, similar to that in BioLLINC [14]. Other platforms have been reported to have even lower publication rates [17]. This may reflect, in part, the relatively low bar for project submission and approval. Perhaps it is more relevant to look at the total number of projects resulting in publications, which is 52 from PLCO and NLST combined, over four years, and is likely to increase substantially in the next few years. The ultimate benefit of these data-sharing platforms, however, goes beyond the number of publications, as wide data sharing enhances public trust in research and encourages collaborative efforts.

## Conclusion

The high degree of interest by the public and research community in the findings of the PLCO and NLST cancer screening trials prompted the NCI to make their data widely available for the purposes of reanalyses of trial findings for confirmation of published results, new analyses of trial-related questions, and other critical research, including cancer etiology and image analysis. Here, we have described a web-based system, CDAS, developed by NCI to facilitate broad sharing of these trials’ data. Our experience with this data-sharing platform over its first four years, including the utilization of the resource, associated costs, and future expansion potential of the site, should help inform the research community about the benefits and drawbacks of this type of data-sharing model.

Given the breadth and diversity of medical research, a variety of data-sharing approaches and platforms are needed to efficiently accommodate the need. A balance must be struck between resource commitments for data sharing and the yield in terms of data requests; what is appropriate for high-profile clinical trials may not be for studies of lesser interest. Arrangements with journals for storing supplementary data files, digital data repositories linked to journal articles, and use of other existing data-sharing platforms, in addition to de novo CDAS-like approaches when appropriate, will all be required to satisfy the increasing demand for data sharing in medical research.

## Supporting information

S1 Text CDAS technical requirements. (DOC)

S2 Text CDAS Data Transfer Agreement. (DOC)

S1 Data Kaplan–Meier analysis of PLCO publications. (XLSX)

S2 Data Kaplan–Meier analysis of NLST publications. (XLSX)

## Abbreviations

BioLINCC: Biologic Specimen and Data Repository Information Coordinating Center
CAD/CAD-X: computer-aided detection or diagnosis
CDAS: Cancer Data Access System
dbGaP: database of Genotypes and Phenotypes
DTA: Data Transfer Agreement
HIPAA: Health Insurance Portability and Accountability Act
ICMJE: International Committee of Medical Journal Editors
IDATA: Interactive Diet and Activity Tracking in AARP
IPD: individual patient data
IRB: institutional review board
LDCT: low-dose computed tomography
NCI: National Cancer Institute
NHLBI: National Heart, Lung, and Blood Institute
NIH: National Institutes of Health
NLST: National Lung Screening Trial
PDS: Project Data Sphere
PHI: protected health information
PI: principal investigator
PII: personally identifiable information
PLCO: Prostate, Lung, Colorectal and Ovarian Cancer Screening Trial
SOAR: Supporting Open Access to Researchers
TCIA: The Cancer Imaging Archive
YODA: Yale Open Data Access
