# Supplementary material for: Data sharing in clinical trials: An experience with two large cancer screening trials
Source: PLoS Med. 2017 May 23;14(5):e1002304. doi: 10.1371/journal.pmed.1002304 (PMC5441574; doi:10.1371/journal.pmed.1002304)
Supplement: S1 Text — (DOC) [file pmed.1002304.s001.doc]

S1 Text. CDAS technical requirements

CDAS is implemented using Django, an open-source web application framework created using the Python programming language. CDAS utilizes ElasticSearch, an open-source Java search engine. PostgreSQL is an open-source database server software used by CDAS for persistent storage. It currently consumes less than 100 megabytes of space. CDAS runs on an Apache HTTP server, an open-source web server.  All products are run on the CentOS Linux platform and are updated continuously.  Files stored on the Linux server include standard datasets available upon request, custom datasets created for special deliveries, dataset documentation, user manuals, and data transfer agreements.  In total, these files currently occupy less than 15 gigabytes. Requested data sets are generally delivered as SAS datasets; however, other options are required upon request.
