# Supplementary material for: Data sharing in clinical trials: An experience with two large cancer screening trials
Source: PLoS Med. 2017 May 23;14(5):e1002304. doi: 10.1371/journal.pmed.1002304 (PMC5441574; doi:10.1371/journal.pmed.1002304)
Supplement: S2 Text — (DOC) [file pmed.1002304.s002.doc]

S2 Text. CDAS Data Transfer Agreement

The National Cancer Institute (NCI) and the Recipient Institution (RECIPIENT) hereby enter into this Agreement for the transfer of data collected in the course of the National Lung Screening Trial (NLST) to RECIPIENT through NCI’s Cancer Data Access System (CDAS). This Agreement is effective and enforceable as of the date of the last signature below (Effective Date).

In consideration of NCI providing DATA to RECIPIENT, RECIPIENT hereby agrees to the following terms and conditions**:**

1. DATA will be used only by RECIPIENT for purpose described in the Research Plan, attached as **Attachment 1**.
2. DATA WILL NOT BE USED TO TREAT OR DIAGNOSE HUMAN SUBJECTS. RECIPIENT will use DATA in compliance with all applicable local, state, and/or federal laws and regulations, including but not limited to those for the protection of human subjects.
3. RECIPIENT must not use DATA for any study other than the approved Research Plan unless RECIPIENT obtains the written consent of NCI by way of a new approved application through CDAS or by written and signed amendment to this Agreement. RECIPIENT grants NCI the right to publicly disclose the Research Plan, including titles, summaries or any other information contained therein, as well as the names and contact information for the investigators conducting the research.
4. NCI authorizes the above-named Recipient Investigators within RECIPIENT’s institution to use the DATA. RECIPIENT may only share DATA with individuals at RECIPIENT’s institution who are necessary to achieve the purpose stated in the Research Plan and who are under the control of one of the above-named Recipient Investigators.  RECIPIENT will advise such individuals of the terms and conditions of usage described herein. RECIPIENT will not share DATA with unauthorized investigators within and outside of Recipient Institution.
5. Access to the DATA by any Collaborating Investigators at other institutions will be governed by the terms of a separately executed agreement between any such institution and NCI.
6. Personally identifiable information will not be provided. If DATA being provided are coded, RECIPIENT will not request the key to the code. RECIPIENT must not attempt to learn the identity of or to contact the human subjects from which DATA were obtained, their physicians, or the collection sites for DATA. In the event that personally identifiable information is inadvertently transferred, RECIPIENT agrees to immediately destroy the personally identifiable information and report the circumstances to NCI.
7. DATA are the property of NCI and are made available as a service to the research community. RECIPIENT will not claim, infer, or imply ownership of DATA or any endorsement of RECIPIENT’S activities or products by the U.S. Government, DHHS, NIH, NCI, or NCI employees.
8. DATA delivered pursuant to this Agreement are experimental in nature. NCI MAKES NO REPRESENTATIONS AND EXTENDS NO WARRANTIES OF ANY KIND, EITHER EXPRESSED OR IMPLIED. THERE ARE NO EXPRESS OR IMPLIED WARRANTIES OF MERCHANTABILITY OR FITNESS FOR A PARTICULAR PURPOSE, OR THAT THE USE OF DATA WILL NOT INFRINGE ANY PATENT, COPYRIGHT, TRADEMARK, OR OTHER PROPRIETARY RIGHTS. Unless prohibited by law, RECIPIENT assumes all liability for claims for damages against it by third parties which may arise from its use, storage or disposal of DATA.
9. RECIPIENT will acknowledge NCI as the source of DATA in all publications and presentations by including language substantially similar to the following: "The authors thank the National Cancer Institute for access to NCI’s data collected by the National Lung Screening Trial. The statements contained herein are solely those of the authors and do not represent or imply concurrence or endorsement by NCI."
10. RECIPIENT must submit a description of each publication resulting from its use of DATA to the following website: <http://biometry.nci.nih.gov/cdas>. RECIPIENT agrees that NCI may publicly disclose this description.
11. This Agreement shall be in effect for three (3) years from the Effective Date. At the end of these three (3) years, if RECIPIENT is still using DATA for the approved Research Plan, RECIPIENT may seek an amendment to extend the term of this Agreement. This Agreement may be terminated by either Party for any reason by providing written notice to the other party at least thirty (30) days prior to the desired termination date. Upon expiration or earlier termination of this Agreement or if RECIPIENT’s use of DATA is complete, RECIPIENT must destroy DATA and provide notice to NCI of the same.
